# Overexpression of miR-27b-3p Targeting Wnt3a Regulates the Signaling Pathway of Wnt/*β*-Catenin and Attenuates Atrial Fibrosis in Rats with Atrial Fibrillation

**DOI:** 10.1155/2019/5703764

**Published:** 2019-04-17

**Authors:** Xiangwei Lv, Jinyi Li, Yisen Hu, Shirong Wang, Chengye Yang, Chengxuan Li, Guoqiang Zhong

**Affiliations:** Departments of Cardiology, First Affiliated Hospital of Guangxi Medical University, Nanning 530021, Guangxi Zhuang Autonomous Region, China

## Abstract

MicroRNAs (miRNAs) are regarded as a potential method for the treatment of atrial fibrillation (AF) although its molecular mechanism remains unknown. We found in our previous study that the level of peripheral blood miR-27b-3p and the expression of atrial tissue CX43 were both significantly downregulated in AF patients. In the present study, we propose and test this hypothesis that overexpression of miR-27b-3p attenuates atrial fibrosis, increases CX43 expression, and regulates the signaling pathway of Wnt/*β*-Catenin by targeting Wnt3a. miR-27b-3p overexpression was induced by rat tail vein injection of adeno-associated virus. Two weeks after transfection of adeno-associated virus, the rat AF model was established by tail vein injection of acetylcholine- (ACh-) CaCl_2_ for 7 days, and 1 ml/kg was injected daily. The incidence and duration of AF were recorded with an electrocardiogram. Cardiac function was monitored by cardiac ultrasound. Serum cardiac enzyme was detected by ELISA. The expression of atrial miR-27b-3 and Wnt3a was assayed by quantitative RT-PCR. Atrial fibrosis was determined by Masson's trichrome staining. Expression of atrial Collagen-I and Collagen-III was tested by the immunohistochemical method. Expression of CX43 was measured by immunofluorescence. The expression of Collagen-I, a-SMA, Collagen-III, TGF-*β*1, CX43, Wnt3a, *β*-Catenin, and p-*β*-Catenin was assayed by western blot. Our results showed that miR-27b-3p overexpression could reduce the incidence and duration of AF, alleviate atrial fibrosis, increase atrial CX43 expression, and decrease the expression of Collagen-I, a-SMA, Collagen-III, TGF-*β*1, Wnt3a, and p-*β*-Catenin. In addition, the results of luciferase activity assay showed that Wnt3a is a validated miR-27b-3p target in HEK 293T cells. Our results provide a new evidence that miR-27b-3p regulates the signaling pathway of Wnt/*β*-Catenin by targeting Wnt3a, which may play an important role in the development of atrial fibrosis and AF.

## 1. Introduction

Atrial fibrillation (AF) is a clinically frequently seen cardiac arrhythmia, and its incidence increases with aging and accompanies heart diseases [1]. In recent years, the incidence and mortality of AF keep increasing, which mainly associate with thrombotic diseases (stroke) and myocardial fibrotic diseases (heart failure). It is a worldwide public health problem that brings a heavy financial burden to society and families [2].

Atrial structural remodeling and electrical remodeling are two important features of AF, which can lead to the occurrence and maintenance of AF. In turn, AF can aggravate atrial structural remodeling and electrical remodeling [3]. Recent studies showed that atrial fibrosis played a crucial role in structural remodeling and that the gap junctional proteins (GJPs) played an important function in atrial electrical remodeling [4, 5]. GJPs mediate electrical coupling of cardiomyocytes. In the mammalian heart, the intercellular channels are comprised of connexins, which allow ion exchange between adjacent cells. In the heart, CX43 is associated with the control of intercellular communication and is the main GJP protein [6]. It has been proven in the animal AF model and AF patients that the atrial tissue CX43 was significantly downregulated at the transcriptional and posttranscriptional levels [7–9].

The signaling pathway of Wnt/*β*-Catenin is a conserved cellular signaling system in animals that regulates cell survival, death, and proliferation and plays an important role in many human diseases [10]. It was found in recent studies that the signaling of Wnt/*β*-Catenin plays an important role in the development and progression of various fibrotic diseases, such as pulmonary fibrosis, liver fibrosis, skin fibrosis, renal fibrosis, and myocardial fibrosis [11, 12]. It was also found that the signaling pathway of Wnt/*β*-Catenin has significant regulation effect on CX43 expression [13, 14].

MicroRNAs (miRNAs) are small, endogenous, noncoding RNAs containing 21-24 nucleotides that, by binding to the 3′ untranslated region (UTR) of their target mRNA, inhibit translation of the target gene or promote its degradation, and the gene expression is regulated at the posttranscriptional level [15]. miRNAs regulate the proliferation and differentiation of the cell by controlling the expression of genes and play an important role in the development of various species [16]. The organization and circulating level of miRNAs are considered being related to the process of cardiovascular diseases. miRNAs play an important role in the structural remodeling and electrical remodeling and are key factors for the incidence of AF [17].

It was found in the AF model of C57BL/6 mice that miR-27b could accelerate the occurrence of AF by inducing atrial fibrosis through the ALK5-targeted regulation of the TGF-*β*1/ALK5/Smad-2/3 pathway [18]. Interestingly, we also found that the level of peripheral blood miR-27b-3p was downregulated. In this study, we investigated the alleviation of atrial fibrosis in AF rats by Wnt3a-targeted (an important member of the Wnt family) regulation of the signaling of Wnt/*β*-Catenin through miR-27b-3p overexpression, a new molecular mechanism at the miRNA level.

## 2. Materials and Methods

### 2.1. Materials and Reagents

The recombinant adeno-associated virus (serotype 9) vector carries rno-miR-27b-3p, AAV2/9-cTNT-miR-27b-3p-GFP (AAV-miR-27b-3p) overexpresses miR-27b-3p, AAV2/9-cTNT-miR-27b-3p-antago-GFP (AAV-miR-27b-3p-antago) underexpresses miR-27b-3p, and AAV2/9-cTNT-GFP (AAV-NC) is a negative control group; c-TNT promoter is specifically expressed in the heart. All reagents were acquired from Hanbio Biotechnology (Shanghai, China). Cardiac troponin T (cTnT) and creatine kinase-MB (CK-MB) ELISA reagent kits were purchased from Cusabio Biotech (MD, USA). Antibodies Collagen-I, Collagen-III, TGF-*β*1, and Wnt3a were supplied by Abcam (Shanghai, China). Antibodies a-SMA, CX43, *β*-Catenin, p-*β*-Catenin, GAPDH, and *β*-Tubulin were obtained from Cell Signaling Technology (Shanghai, China). Masson's trichrome reagent kit was purchased from Solarbio (Beijing, China). BCA protein quantification kit and the secondary antibody were purchased from Beyotime (Changsha, China).

### 2.2. Animal and Ethics

Sprague-Dawley male rats, weighing 200-250 g and 8 weeks old, were raised under standard experimental conditions, 20°C-25°C, 50%-60% RH, and 12 L/12 D photoperiod. The experimental rats were supplied clean drinking water and food *ad libitum*. The rats were purchased from the experimental animal center of Guangxi Medical University (Execute no. SCXK [Gui] 2014-0003). The experiments were subject to approval by the Ethics Committee of Experimental Animals, Guangxi Medical University.

### 2.3. Animal Experiment

40 rats were divided into five groups randomly: (1) sham group: rats in this group were injected with 0.9% of normal saline daily via the tail vein at 1 ml/kg for 7 days; (2) AF group [19]: rats in this group were injected with acetylcholine- (ACh-) CaCl_2_ (60 *μ*g/ml ACh and 10 mg/ml CaCl_2_) daily via the tail vein at 1 ml/kg for 7 days; (3) AAV-NC group: rats in this group were injected with AAV-NC (2 × 10^11^ vector genome (vg) particles/per rat) via the tail vein. 14 days after injection, these rats were injected with acetylcholine- (Ach-) CaCl_2_ (60 *μ*g/ml ACh and 10 mg/ml CaCl_2_) daily via the tail vein at 1 ml/kg for 7 days; (4) AAV-miR-27b-3p group: rats in this group were injected with AAV+miR-27b-3p (2 × 10^11^ vector genome (vg) particles/per rat). 14 days after injection, these rats were injected with acetylcholine- (ACh-) CaCl_2_ (60 *μ*g/ml ACh and 10 mg/ml CaCl_2_) daily via the tail vein at 1 ml/kg for 7 days; and (5) AAV-miR-27b-3p-antago group: rats in this group were injected with AAV-miR-27b-3p-antago (2 × 10^11^ vector genome (vg) particles/per rat) via the tail vein. 14 days after injection, these rats were injected acetylcholine- (ACh-) CaCl_2_ (60 *μ*g/ml ACh and 10 mg/ml CaCl_2_) daily via the tail vein at 1 ml/kg for 7 days.

### 2.4. Electrocardiogram Recording and Analysis

Before tests, rats were paralyzed with pentobarbital sodium (40 mg/kg). The limb leads of a 12-lead electrocardiogram were inserted into the animal's four limbs and the nonsinus rhythm was recorded and excluded. Seven days after model construction, the rats were subjected to electrocardiogram test again, and the incidence and duration of AF were recorded.

### 2.5. RT-PCR Assay of Atrial Tissues

Rat hearts were removed and the left atrial tissue was isolated. Total RNA and miRNA were extracted using RNAeasy Mini Kit (Qiagen, Netherlands) and miRcute miRNA isolation kit (TIANGEN Biotech, China) following the manufacturer's instruction, respectively. Quantitative detection of total RNA and miRNA was performed by using a NanoDrop 2000 spectrophotometer (Thermo Scientific, USA). Further detection of miR-27b-3p and Wnt3a levels and RNA reverse transcription to cDNA using miRcute Plus miRNA First-Strand cDNA Synthesis Kit (TIANGEN Biotech, China) and RevertAid First Strand cDNA Synthesis Kit (Thermo Scientific, USA) were performed according to their respective protocols. The primers of miR-27b-3p, Wnt3a, U6, and *β*-actin were designed and synthesized by TaKaRa (Kyoto, Japan) (Table 1). The RT-PCR assay for miR-27b-3p and Wnt3a was performed with the ABI 7500 Real-Time (RT) PCR System (Applied Biosystems, USA) with miRcute Plus miRNA qPCR Detection Kit (SYBR Green) (TIANGEN, China) and Fast SYBR Green Master Mix Kit (Applied Biosystems, USA) by the manufacturer's instructions, respectively. Fold changes in the miR-27b-3p and Wnt3a level were calculated using 2^-△△Ct^ methods and U6 or *β*-actin used as an internal control, respectively.

### 2.6. miR-27b-3p Targeted Gene Prediction and Luciferase Reporter Gene Assays

**Figure 1:**
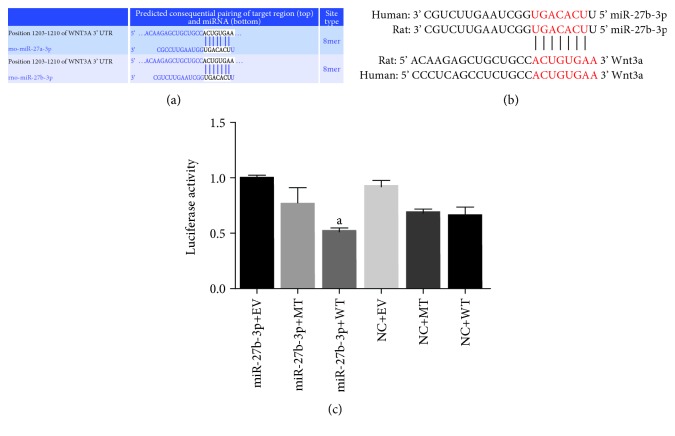
miR-27b-3p negatively regulated the expression of Wnt3a (*n* = 6 for each group). (a) and (b) predicted binding sequence of miR-27b-3p and Wnt3a in rat and human. (c) Quantitative analysis of luciferase reporter gene results by Prism 6.0 software. Note that ^a^*P* < 0.05 against the miR-27b-3p+MT group.

We used TargetScan 7.2 (http://www.targetscan.org/) to predict and analyze the binding sites of miR-27b-3p and Wnt3a as shown in Figures 1(a) and 1(b). To test if Wnt3a is the target gene of miR-27b-3p, we first constructed the 3′ UTR of wild-type (WT) Wnt3a and cloned it to the downstream pMIR plasmid vector of the dual-luciferase reporter gene. Secondly, we constructed the 3′ UTR of mutant-type (MT) Wnt3a, which was replaced by the 5 bp synthesized at the seed region of miR-27b-3p and then inserted in the same plasmid vector. HEK 293T cells at their logarithmic growth phase were collected and transferred to the 96-well plate at 2 × 10^4^ cells/well. The cells were incubated at 37°C and 5% of CO_2_ in a cultivation chamber. miR-27b-3p mimics or miR-27b inhibitor was transfected for 48 h according to the user's manual of Lipofectamine®3000 Transfection Reagent. The luciferase activity was tested with the dual-luciferase reporter system (Promega, WI, USA).

### 2.7. Ultrasonic Cardiogram Analysis

Rats were paralyzed after successful model construction; left ventricular ejection fraction (EF), left ventricular fractional shortening (FS), left ventricular end-systolic diameter (LVESD), and left ventricular end-diastolic diameter (LVEDd) of these animals were analyzed with the MS400 biological signal analysis system (Longfeida Technology Co. Ltd., Shandong, China). The mean value of three cardiac cycles was used for statistical analysis.

### 2.8. Myocardial Enzyme Test

Arterial blood was collected at the apex with heparin anticoagulation tube and then centrifugated by 8000g at 4°C for 10 min. Supernatant was collected to determine the levels of cTnT and CK-MB with ELISA reagent kit according to manufacturer's instruction.

### 2.9. Atrial Tissue Fibrosis Study

The hearts of the rats were quickly removed under anesthesia and the blood was washed with PBS. The left atrial tissue was isolated and fixed with 4% of paraformaldehyde. The sample was imbedded into paraffin and cut into 3 mm thick slices. The slices were stained with Masson's trichrome staining and observed under an optical microscope (CKX41, Olympus, Tokyo, Japan). The samples were observed at × 400, and three visual fields were tested in each sample. Results were analyzed with Image-Pro 6.0 software.

### 2.10. Immunohistochemical Analysis of Atrial Tissue

3 mm thick sections were incubated overnight at 4°C using Collagen-I and Collagen-III primary antibody (dilution to 1 : 150). After incubation with the primary antibodies, the sections were washed 3 times at room temperature for 5 min each time and it was incubated with a secondary antibody for 2 h at room temperature. The samples were observed under the optical microscope (CKX41, Olympus, Tokyo, Japan) at ×400, and three visual fields were tested randomly in each sample. Results were analyzed with Image-Pro 6.0 software.

### 2.11. Immunofluorescence Analysis of Atrial Tissue

To determine the expression site of CX43, every group was subjected to immunofluorescence test. Sections 3 mm thick were taken and blocked with 10% serum for 1 h. Colocalization detection was then performed by incubating overnight at 4°C with CX43 primary antibody (diluted to 1 : 150). After the incubation of the primary antibody, the sections were washed 3 times at room temperature for 5 min each time. Then, it was incubated with a secondary antibody for 2 h at room temperature. DAPI staining was performed for 10 min before the samples were observed using an optical microscope (CKX41, Olympus, Tokyo, Japan), at ×400 and three visual fields were tested randomly in each sample. Results were analyzed with Image-Pro 6.0 software.

### 2.12. Western Blot Assay of Atrial Tissue

Equal amount of left atrial tissue was collected and lysed in the RIPA buffer of phenylmethylsulfonylfluoride (PMSF). Protein content was quantified with the BCA reagent kit and then subjected to SDS-PAGE. The protein was subsequently transferred to the PVDF membrane. The PVDF was incubated by Collagen-I, a-SMA, Collagen-III, TGF-*β*1, CX43, Wnt3a, *β*-Catenin, p-*β*-Catenin, GAPDH, and *β*-Tubulin for 6 h. Concentration of the primary antibodies were 1 : 2000 for Collagen-I, Collagen-III, TGF-*β*1 and *β*-Catenin; 1 : 500 for p-*β*-Catenin; and 1: 1000 for a-SMA, CX43, Wnt3a, GAPDH and *β*-Tubulin. Concentration of the secondary antibody was 1 : 14000 and it was incubated for 2 h at room temperature. The signal was tested with the chemiluminescence system (Amersham Pharmacia).

### 2.13. Data and Statistical Analysis

Data are expressed as mean ± standard deviation and data were analyzed using Student's t-test or one-way ANOVA. Statistical analysis was performed using Prism 6.0 software package. *P* < 0.05 was considered as statistical significance.

## 3. Results

### 3.1. miR-27b-3p Overexpression Reduced the Incidence and Duration of AF Rats

**Figure 2:**
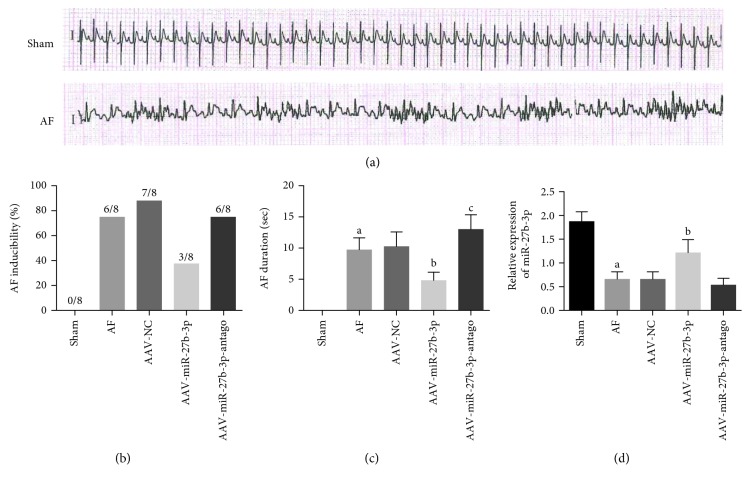
miR-27b-3p overexpression could reduce the incidence and duration of AF (*n* = 8 for each group). (a) Successful establishment of the AF rat model. (b) Incidence of AF. (c) Duration of AF. (d) The level of miR-27b-3. Note that ^a^*P* < 0.05 against the sham group; ^b^*P* < 0.05 against the AF group; ^c^*P* < 0.05 against the AF group.

As shown in Figure 2(a), regular P wave was observed in the sham group according to electrocardiography, suggesting normal sinus rhythm. Disappeared P wave and irregular R-R interphase change in the AF group were observed, suggesting successful construction of an AF model. As shown in Figures 2(b) and 2(c), decreased incidence and shortened duration of AF were observed in the AAV-miR-27b-3p group as compared to the AF group. Furthermore, the results of RT-PCR assay showed that the miR-27b-3p level in the AF group decreased significantly compared with the sham group. However, the level of miR-27b-3p in the AAV-miR-27b-3p group increased significantly compared with the AF group (Figure 2(d)).

### 3.2. Effects of miR-27b-3p Overexpression on Cardiac Function of AF Rats

**Figure 3:**
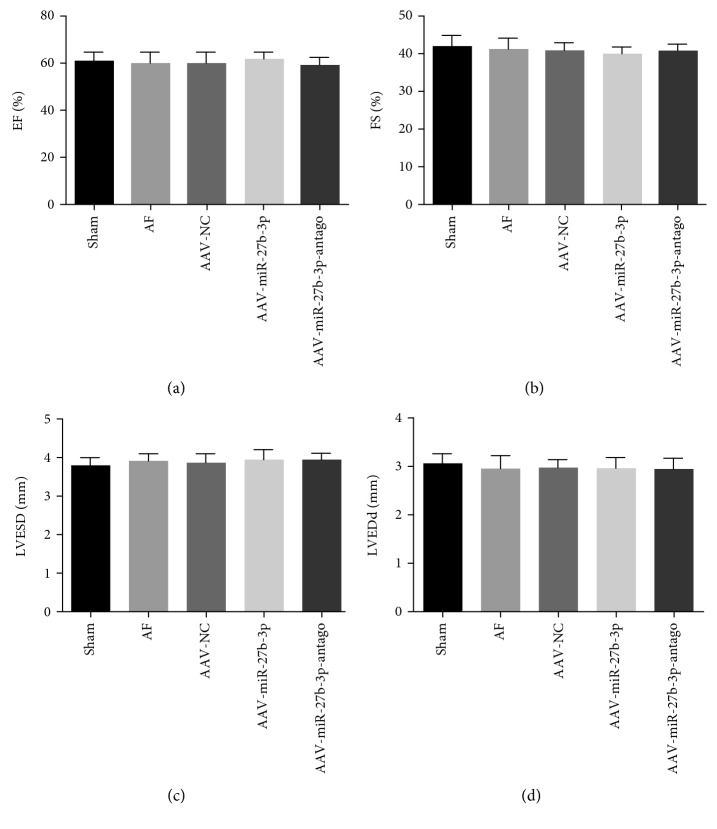
miR-27b-3p overexpression did not have a significant effect on the cardiac function (*n* = 8 for each group). (a) EF: left ventricular ejection fraction. (b) FS: left ventricular fractional shortening. (c) LVESD: left ventricular end-systolic diameter. (d) LVEDd: left ventricular end-diastolic diameter.

The results of cardiac ultrasound showed no significant changes in the cardiac function in the AF group, AAV-NC group, AAV-miR-27b-3p group, and AAV-miR-27b-3p-antago group compared to the sham group as shown in Figures 3(a)–3(d).

### 3.3. Effects of miR-27b-3p Overexpression on Myocardial Enzyme of AF Rats

**Figure 4:**
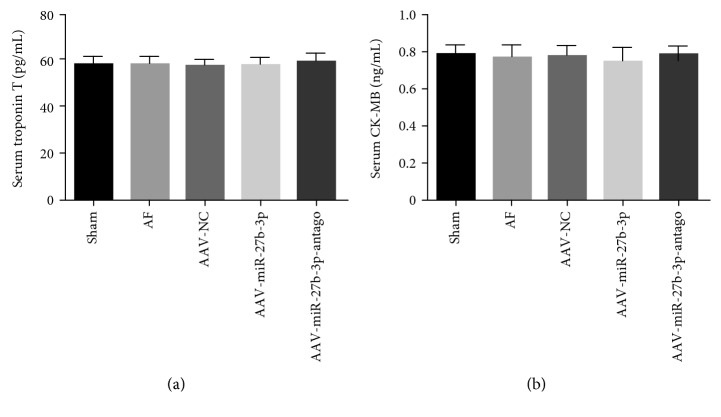
miR-27b-3p overexpression did not have a significant effect on myocardial enzyme (*n* = 8 for each group). (a) Serum level of cTnT. (b) Serum level of CK-MB.

As shown in Figures 4(a) and 4(b), the results of the ELISA test suggested no significant changes of myocardial enzyme in the AF group, AAV-NC group, AAV-miR-27b-3p group, and AAV-miR-27b-3p-antago group compared to the sham group.

### 3.4. miR-27b-3p Overexpression Alleviated Atrial Fibrosis of AF Rats

**Figure 5:**
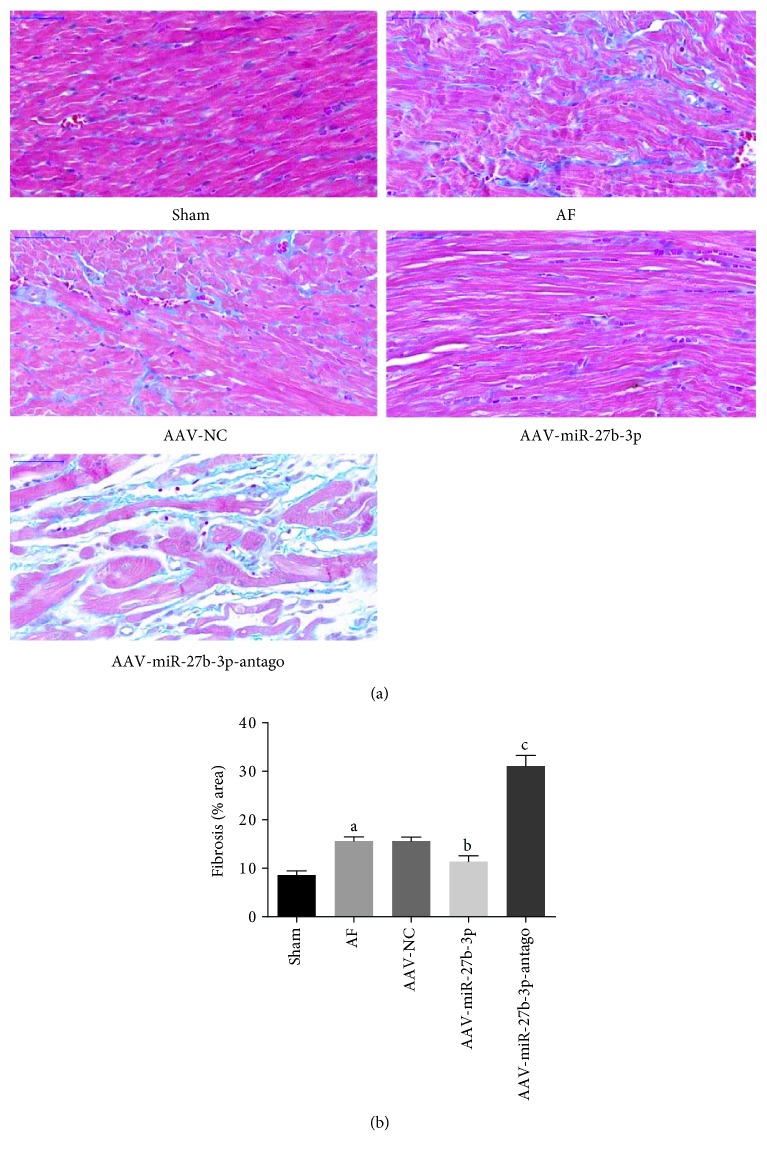
Effects of miR-27b-3p overexpression on atrial fibrosis (*n* = 8 for each group). (a) Representative images of Masson's trichrome staining (×400) in the left atrial tissues of each group. (b) The degree of myocardial fibrosis was analyzed by the Image-Pro 6.0 software. Note that ^a^*P* < 0.05 against the sham group; ^b^*P* < 0.05 against the AF group; ^c^*P* < 0.05 against the AF group.

**Figure 6:**
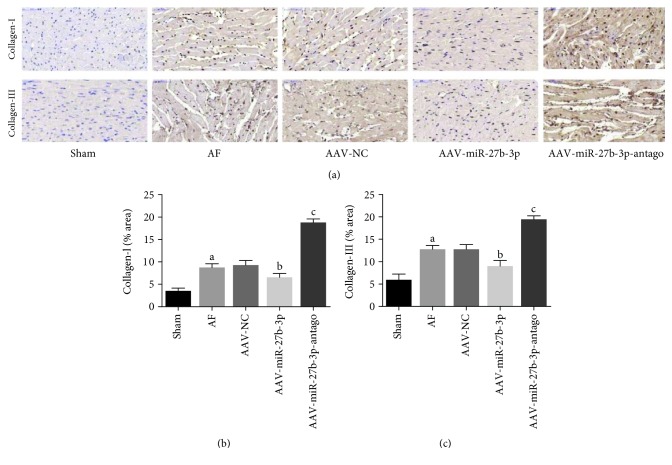
Effects of miR-27b-3p overexpression on atrial fibrosis (*n* = 8 for each group). (a) Representative images of immunohistochemistry staining (×400) in the left atrial tissues of each group. (b, c) The quantitative analyses by the Image-Pro 6.0 software. Note that ^a^*P* < 0.05 against the sham group; ^b^*P* < 0.05 against the AF group; ^c^*P* < 0.05 against the AF group.

**Figure 7:**
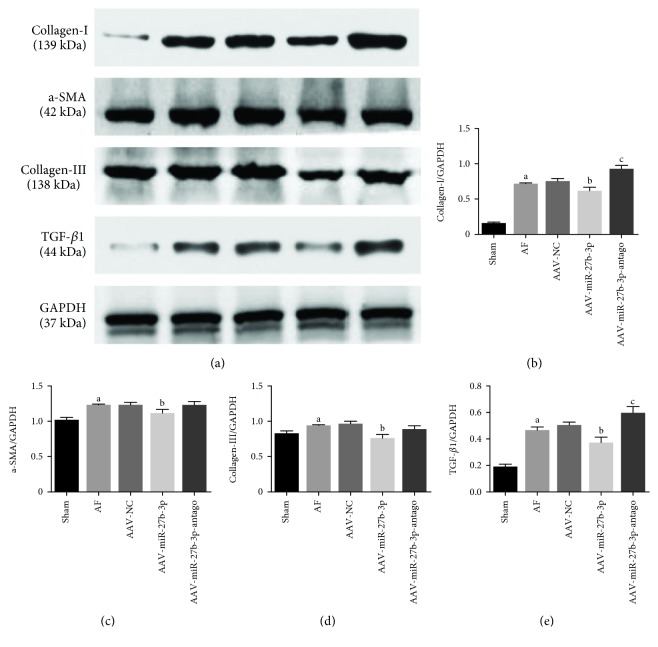
Effects of miR-27b-3p overexpression on atrial fibrosis (*n* = 8 for each group). (a) Expression level of markers of fibrosis in the myocardial tissues detected using western blot and quantitative analyses by the Image-Pro 6.0 software (b–e). Note that ^a^*P* < 0.05 against the sham group; ^b^*P* < 0.05 against the AF group; ^c^*P* < 0.05 against the AF group.

1. Masson's trichrome staining test showed that the atrial fibrosis level of the AF group increased significantly compared with the sham group, while it was reduced significantly in the AAV+miR-27b-3p group compared with the AF group. However, a higher atrial fibrosis level was observed in the AAV-miR-27b-3p-antago group compared with the AF group as shown in Figures 5(a) and 5(b)
2. Results of immunohistochemical test showed that the expression level of Collagen-I and Collagen-III in the AF group increased significantly compared with the sham group, in which their expression levels decreased significantly in the AAV-miR-27b-3p group compared with the AF group. However, the expression level of Collagen-I and Collagen-III in the AAV-miR-27b-3p-antago group increased significantly compared with the AF group (Figures 6(a)–6(c))
3. Results of western blot test showed that the expression of Collagen-I, a-SMA, Collagen-III, and TGF-*β*1 in the AF group increased significantly compared with the sham group, while their expression decreased significantly in the AAV-miR-27b-3p group compared with the AF group. However, the expression of Collagen-I and TGF-*β*1 in the AAV-miR-27b-3p-antago group increased significantly compared with the AF group (Figures 7(a)–7(e))

### 3.5. miR-27b-3p Overexpression Increased the Expression of Atrial CX43 in AF Rats

**Figure 8:**
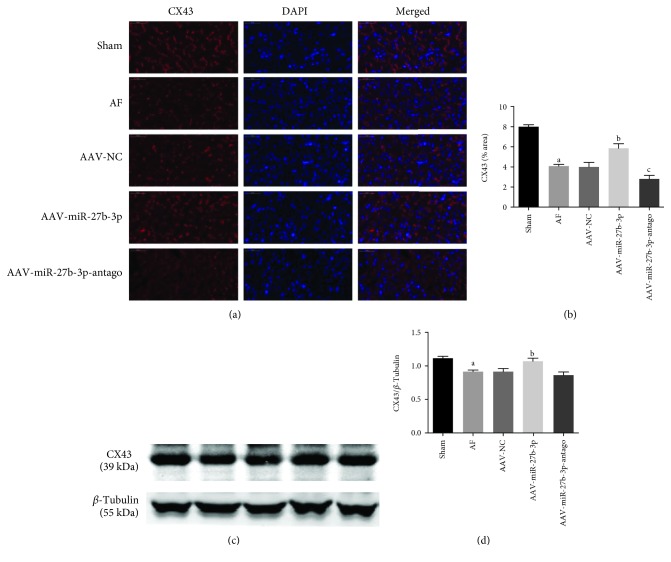
miR-27b-3p overexpression increased the expression of atrial CX43 (*n* = 8 for each group). (a) Representative images from the immunofluorescence staining (×400) in left atrial tissues. Red fluorescence represents CX43; blue fluorescence represents nuclei of total cardiac myocytes. (b) The quantitative analyses by the Image-Pro 6.0 software. (c) Expression of CX43 in the atrial tissues detected using western blot and quantitative analyses by the Image-Pro 6.0 software (d). Note that ^a^*P* < 0.05 against the sham group; ^b^*P* < 0.05 against the AF group; ^c^*P* < 0.05 against the AF group.

As shown in Figures 8(a) and 8(b), the results of the immunofluorescence test showed that the expression of CX43 in the AF group decreased significantly compared with that in the sham group, while it was increased significantly in the AAV-miR-27b-3p group compared with the AF group. However, the expression of CX43 in the AAV-miR-27b-3p-antago group decreased significantly compared with that in the AF group. Furthermore, the results of the western blot test showed that the expression of CX43 in the AF group decreased significantly compared with that in the sham group, while it was increased significantly in the AAV-miR-27b-3p group compared with the AF group (Figures 8(c) and 8(d)).

### 3.6. miR-27b-3p Regulates the Expression of Wnt/*β*-Catenin Signaling Pathway by Targeting Wnt3a

**Figure 9:**
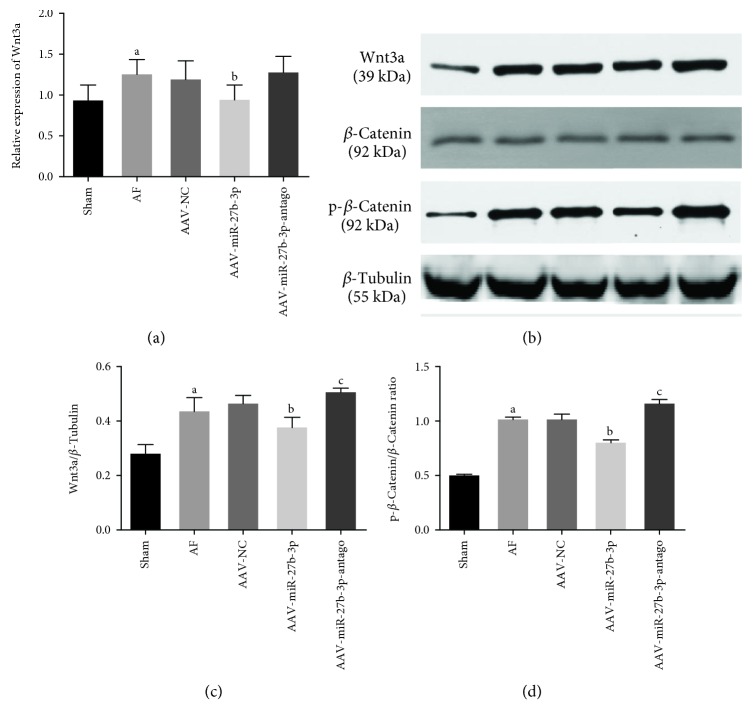
miR-27b-3p overexpression attenuated the expression of the Wnt/*β*-Catenin signaling pathway (*n* = 8 for each group). (a) The level of Wnt3a. (b) Expression of Wnt3a and p-*β*-Catenin in the atrial tissues detected using western blot and quantitative analyses by the Image-Pro 6.0 software (c, d). Note that ^a^*P* < 0.05 against the sham group; ^b^*P* < 0.05 against the AF group; ^c^*P* < 0.05 against the AF group.

1. In order to further investigate the mechanism that miR-27b-3p overexpression alleviated atrial fibrosis, we used the online software TargetScan (http://www.targetscan.org/) to predict and analyze if Wnt3a was the target gene of miR-27b-3p (Figures 1(a) and 1(b)). As shown in Figure 1(c), results of luciferase reporter assays showed that the luciferase activity in the miR-27b-3p+WT group was significantly lower than that in the miR-27b-3p + MT group. Wnt3a is a validated target gene of miR-27b-3p in HEK 293T cells
2. As shown in Figure 9(a), the Wnt3a level by RT-PCR assay in the AF group showed to be increased significantly compared with that in the sham group, while it was decreased significantly in the AAV-miR-27b-3p group compared with the AF group. Furthermore, the results of the western blot test showed that the expression of Wnt3a and p-*β*-Catenin in the AF increased significantly compared with that in the sham group, while their expression in the AAV-miR-27b-3p group decreased significantly compared with that in the AF group. However, the expression of Wnt3a and p-*β*-Catenin in the AAV-miR-27b-3p-antago group increased significantly compared with that in the AF group as shown in Figures 9(b)–9(d)

## 4. Discussion

### 4.1. miRNAs Participate into the Occurrence of AF

The pathogenesis of AF is rather complicated, and current research suggests that it mainly involves structural remodeling, electrical remodeling, metabolic abnormalities, and neurohormone and molecular changes [20, 21]. In recent years, studies have confirmed that miRNAs are involved in the occurrence and development of AF and play an important role in regulating atrial structural remodeling and electrical remodeling [22]. Studies with AF patients and AF model animals suggested that atrial structural remodeling is a crucial factor for the occurrence and development of AF, whereas fibrosis is an important feature of structural remodeling. Abnormal synthesis, degradation, and deposition of atrial collagen could promote atrial fibrosis and structural remodeling [23, 24]. Some miRNAs such as miR-1, miR-21, miR-27b, miR-29, miR-30, and miR-590 have been proven to regulate the process of atrial structure remodeling [17, 18]. Atrial electrical remodeling plays an identically important role in the occurrence and development of AF. Electric change in atrial muscle may lead to partial functional loss of the ion channel, whereas abnormal electric current change of the ion channel is related to intracellular transportation of Ca^2+^ and K^+^, which further affects the remodeling and structural change of GJPs. Such changes may promote the maintenance of AF. In recent years, some miRNAs such as miR-25, miR-106, miR-223, miR-133, and miR-328 have been proven to be related to the electrical remodeling [25–28].

In the study, we constructed successfully the acetylcholine- (ACh-) CaCl_2_-induced AF rat model. Our results showed that miR-27b-3p expression was downregulated in the left atrium of the AF rat model, while the markers of fibrosis Collagen-I, a-SMA, and Collagen-III were upregulated, and the degree of atrial fibrosis increased significantly. In contrast, miR-27b-3p overexpression could alleviate atrial fibrosis and reduce the susceptibility and duration of AF. However, there was no significant change in the cardiac function and myocardial injury markers (cTnT and CK-MB). Based on the pathophysiology perspective, we hypothesize that the animal's cardiac function is still in the compensatory period, further speculating that this is related to the duration of AF and the stage of development of AF. In the next stage, we will extend the duration of AF and further observe the changes in the cardiac function and myocardial injury markers in experimental animals.

### 4.2. Wnt/*β*-Catenin Signaling Pathway Regulates Fibrosis and CX43 Expression

Wnt protein family is a secreted lipid-modified glycoprotein that can regulate a lot of intracellular signaling transduction cascade. *β*-Catenin is a key factor in the signal transduction, mediating the canonical Wnt/*β*-Catenin pathway. It regulates the transcription of fibrosis-related genes in the Wnt pathway such as fibronectin, matrix metalloproteinases-7 (MMP-7), plasminogen activator inhibitor-1 (PAI-1), twist, and snail [29, 30]. In addition, Wnt/*β*-Catenin and TGF-*β* signaling pathway regulates the fibrosis process in a cross-talk pattern [31]. Therefore, it is not surprising that the signaling pathway of Wnt/*β*-Catenin regulates the fibrotic diseases. It was found in recent years that there is a cross-talk function between the signaling pathway of Wnt/*β*-Catenin and CX43 [32]; as an upstream regulatory factor, CX43 regulates negatively the canonical Wnt/*β*-Catenin pathway. Overexpression of CX43 can reduce the transcriptional activity of *β*-Catenin protein in lithium-stimulated neonatal rat cardiomyocytes [33]. In addition, CX43 knockout in bone cells led to the accumulation of *β*-Catenin protein and increased the expression of target genes of the signaling pathway of Wnt/*β*-Catenin [34]. It was also found that CX43 is the downstream target gene of the canonical Wnt/*β*-Catenin pathway and subjected to its positive regulation and translation [32]. In cardiomyocytes, the signaling pathway of Wnt/*β*-Catenin can upregulate CX43 expression, and activation of Wnt/*β*-Catenin signaling pathway can upregulate CX43 expression in mouse embryonic stem cells and further induce cardiac differentiation [35]. In addition, in HL-1 cells, inhibition of *β*-Catenin could block mesenchymal stem cell- (MSC-) induced upregulation of CX43 and improve cardiac transduction, indicating the MCSs could alleviate the incidence of arrhythmia by activating the canonical signaling pathway of Wnt/*β*-Catenin [36].

In the present study, we found that Wnt3a, p-*β*-Catenin, and TGF-*β*1 expression in the left atrium were significantly increased, and CX43 expression was significantly decreased. Overexpression of miR-27b-3p reduced Wnt3a, p-*β*-Catenin, and TGF-*β*1 expression levels and increased CX43 expression. We therefore speculate that there may be a regulatory effect between miR-27b-3p and the signaling pathway of Wnt/*β*-Catenin and CX43. No matter if CX43 negatively regulates the canonical signaling pathway of Wnt/*β*-Catenin as an upstream regulatory factor, or as a downstream target gene of the canonical signaling pathway of Wnt/*β*-Catenin, and is subjected to its positive or negative regulation in term of transcription and translation, it was obvious condition-dependent according to literature reports. Therefore, we speculated that the regulatory relationship between the signaling pathway of Wnt/*β*-Catenin and CX43 depends on disease specificity and tissue specificity, as well as different stages of disease progression.

### 4.3. miR-27b-3p Regulation of the Signaling Pathway of Wnt/*β*-Catenin Is a New Mechanism for the Occurrence of AF

In recent years, studies have found that miRNAs participate in the process of various fibrotic diseases by regulating the signaling pathway of Wnt/*β*-Catenin. For instance, miR-708-5p-, miR-217-5p-, and miR-499-5p-mediated regulation of the Wnt/*β*-Catenin signaling pathway is probably a potential pathophysiological mechanism of arrhythmogenic cardiomyopathy (AC) [37]; miR-375 targets frizzled 8 to regulate rat alveolar epithelial cell transdifferentiation via the signaling pathway of Wnt/*β*-Catenin [38]. In another study, it was found that miR-378a-3p could inhibit the activity of hepatic stellate cells (HSCs) through the signaling pathway of Wnt/*β*-Catenin by targeting Wnt10a [39]; miR-29a could alleviate liver fibrosis through regulation of the signaling of Wnt/*β*-Catenin [40]. Furthermore, the miR-27 family also plays an important role in the development and progression of various diseases by regulating the Wnt/*β*-Catenin signaling pathway [41–43]. miR-27a regulates the activity of Wnt/*β*-catenin pathway and plays an important role in the differentiation of laryngeal carcinoma by targeting GSK-3*β* [44]; miR-27 can recruit *β*-catenin to accumulate in the nucleus by targeting APC and activate Wnt/*β*-catenin pathway to promote odontoblastic differentiation [45].

**Figure 10:**
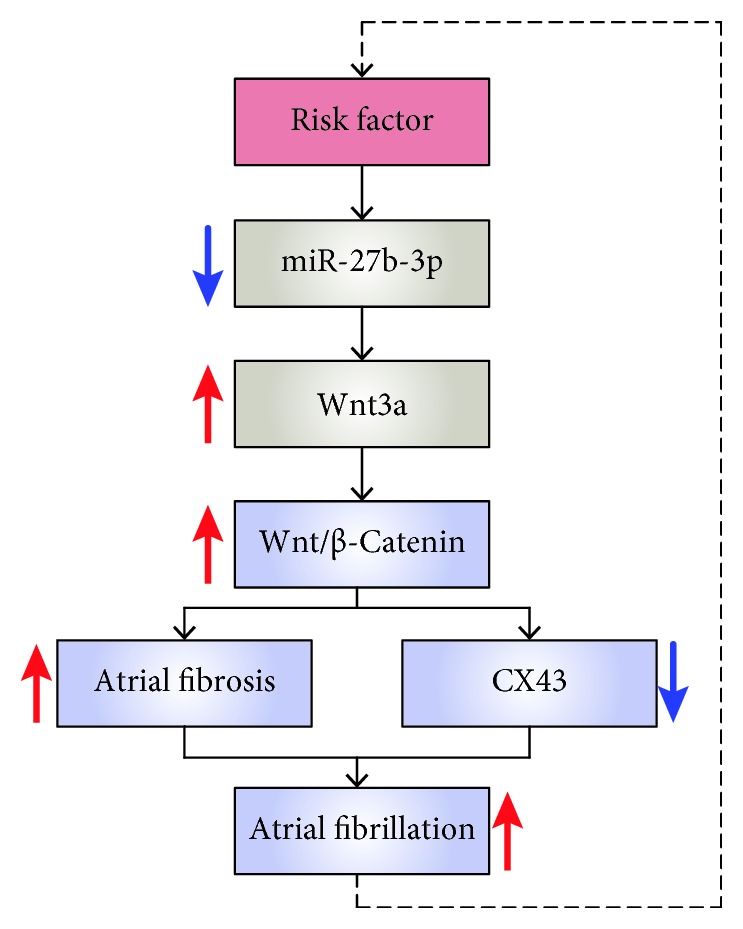
The molecular mechanism diagram showed that miR-27b-3p played an important role in atrial fibrosis and the occurrence of AF by regulation of the signaling pathway of Wnt/*β*-Catenin through targeted Wnt3a.

In the present study, to further investigate the mechanism that miR-27b-3p overexpression alleviates atrial fibrosis and reduces the incidence and duration of AF, we proved that Wnt3a is a validated target gene of miR-27b-3p using bioinformatics and the luciferase reporter assays. We found that miR-27b-3p overexpression could reduce the expression of Wnt3a and p-*β*-Catenin. We therefore believed that miR-27b-3p could negatively regulate the expression of Wnt3a, further affecting the activity of the Wnt/*β*-Catenin signaling pathway. It is further speculated that miR-27b-3p overexpression probably has alleviated the atrial fibrosis of AF rats through the signaling pathway of Wnt/*β*-Catenin. Furthermore, our results indicated that, as summarized schematically in Figure 10, miR-27b-3p regulation of Wnt/*β*-Catenin signaling pathway through targeting Wnt3a could promote atrial fibrosis and the incidence of AF, which is a new molecular mechanism at the miRNA level for AF.

## 5. Conclusion

Our results supplied a new evidence that downregulated miR-27b-3p could promote atrial fibrosis and occurrence of AF by regulation of the signaling pathway of Wnt/*β*-Catenin through targeting Wnt3a. Therefore, miR-27b-3p probably supplies a new valuable target for gene therapy of AF.

## Figures and Tables

**Table 1:** Primer sequences for RT-PCR.

Primer/probe	Sequences
miR-27b-3p	F: 5′-CTCAACTGGTGTCGTGGAGTCGGCAATTCAGTTGAGGCAGAACT-3′
	R: 5′-ACACTCCAGCTGGGTTCACAGTGGCTAAG-3′
Wnt3a	F: 5′-ACCATGTTCGGGACCTATTCCA-3′
	R: 5′-GCCTGTAGCATCTCGCTTCCA-3′
U6	F: 5′-CTCGCTTCGGCAGCACA-3′
	R: 5′-AACGCTTCACGAATTTGCGT-3′
*β*-Actin	F: 5′-TGCTATGTTGCCCTAGACTTCG-3′
	R: 5′-GTTGGCATAGAGGTCTTTACGG-3′

## Data Availability

The data used to support the findings of this study are available from the corresponding author upon request.
